# Treatment of the Distal Forearm Fracture by Volar Dual Window Approach

**DOI:** 10.3390/life14080972

**Published:** 2024-08-02

**Authors:** Wei-Ting Wang, Chiang-Sang Chen

**Affiliations:** 1 Department of Orthopedic Surgery, Far Eastern Memorial Hospital, New Taipei City 220, Taiwan; femh91919@femh.org.tw; 2 Department of Materials and Textiles, Asia Eastern University of Science and Technology, New Taipei City 220, Taiwan; 3 Department of Dentistry, National Yang Ming Chiao Tung University, Taipei 30010, Taiwan

**Keywords:** distal forearm fracture, dual window approach, unstable distal radioulnar joint, osteosynthesis

## Abstract

Background: Distal forearm fractures were defined as distal radius fractures with concomitant distal ulna fractures, except ulna styloid fractures. Distal forearm fractures are common among geriatric populations, particularly those with osteoporosis. Conventionally, distal forearm fractures are reduced by a double incision approach; however, malreduction and instability of the distal radioulnar joint were not uncommon. We introduced a modified volar dual window approach to treat the distal forearm fracture and evaluate the functional outcomes and complications. Methods: From January 2020 to June 2023, 13 patients with distal forearm fractures underwent open reduction by the modified dual window approach with locking plate fixation. After surgery, splints were applied for two weeks, and the patients underwent postoperative hand therapy for three months. The mean Quick Disabilities of the Arm, Shoulder, and Hand scores, range of motions, grip strength, postoperative radiographic parameters, and complications data were collected. Results: The mean follow-up period was 12.1 months, and the mean age was 52.3 years. Average wrist flexion was 67°, extension 69°, pronation 81°, and supination 79°. Grip strength was 28.3 ± 11.5 kg, which was 88% of the uninjured opposite side. The Visual Analog Scale score during activities was recorded as 0.5 ± 0.9. The mean Quick Disabilities of the Arm, Shoulder, and Hand score was 14 ± 11.5. The postoperative radiographic parameters were as follows: radial height: 10.8 ± 1.7 mm, radial inclination: 22.6 ± 3.7°, volar tilting: 4.0 ± 3.9°, and ulnar variance: −0.4 ± 1.4 mm. All the patients achieved bone union at the final follow-up. Two patients underwent ulnar implant removal due to irritation symptoms. Neither infection, nor neurovascular injury, nor malreduction developed in these patients. Conclusions: The modified volar dual window approach can achieve good wrist function and distal forearm fracture reduction without increasing neurovascular or wound healing complications. This method is an alternative approach for distal forearm fracture, especially in comminuted distal ulna fracture or distal radioulnar joint incongruity.

## 1. Introduction

Isolated distal ulna head or neck fractures are rare [1], and approximately 5.6% of distal radius fractures (DRFs) were combined with distal ulna fractures (DUFs) [2]. A recent Swedish study reports an incidence rate of 74 per 100,000 person–years for distal ulna fractures, whether accompanied by a radius fracture or not. While extensive research has been dedicated to treating distal radius fractures, distal ulna fractures have garnered considerably less attention. These fractures, particularly those excluding the ulnar styloid, are relatively rare. Consequently, robust evidence is lacking to guide the treatment of associated distal ulna fractures [3]. In addition, the primary concern is that the distal radioulnar joint (DRUJ) may be affected if the distal forearm fracture (DFF) is unstable, potentially resulting in instability of the DRUJ, reduced range of motion, malunion, and diminished grip strength if left untreated [4,5,6]. Currently, there is yet to be a consensus on the optimal treatment of DUFs. Treating distal ulna metaphyseal fractures should follow the management of DRFs. Cast immobilization is the preferred method for stable DUF. However, if the fracture shows malalignment or instability of the DRUJ, fixing the DUF should be considered [7].

The distal ulna is the rotational axis for the distal radius during forearm movement, playing a crucial role in wrist biomechanics and load distribution. In a neutral rotational position, the ulnocarpal joint supports approximately 20% of the load on the wrist. This load increases when the forearm is pronated or during gripping actions, accompanied by a relative lengthening of the ulna [8]. For the treatment of DUFs, few studies with limited evidence were addressed [9,10]. The “Arbeitsgemeinschaft für Osteosynthesefragen” (AO) has developed a detailed and user-friendly classification system [11]. However, it neither predicts outcomes nor guides treatment decisions. The Biyani classification, while regarded as the easiest to use and most suitable for distal ulna fractures, does not accommodate fractures extending into the diaphysis [12]. As a result, neither classification system holds significant clinical value [13]. The necessity of fixing DUFs remains controversial across all age groups of patients with DFFs. Nonetheless, conservative treatment of DUF with cast immobilization has proven successful following the rigid fixation of DRF [1,14].

However, fixing DUF can help restore the anatomical alignment and congruency of the DRUJ, facilitating early mobilization. This is essential because wrist joint incongruity results in osteoarthritis in over 90% of patients. As a result, multiple reports suggest assessing DRUJ stability after DRF fixation and recommending DUF fixation if instability is observed [1,7,14]. In addition, several studies recommend operative treatment for unstable DUFs to provide stability and enhance patient satisfaction [15].

Recently, plate osteosynthesis has been widely used to fix DUFs, ensuring stability and allowing for early mobilization, thus facilitating a return to pre-injury status [16]. Most procedures have been performed using traditional approaches, such as the modified Henry’s approach for DRF and the direct lateral approach to the DUF [7,15]. While osteosynthesis shows promise, the traditional direct lateral approach has several drawbacks, including the risk of dorsal cutaneous branch of the ulnar nerve (DCBUN) injury, implant impingement, malreduction, implant failure, and arthrosis, particularly in cases of comminuted metaphyseal fractures or fractures involving the DRUJ [17,18].

We propose a new modified technique based on the modified Henry’s approach to DFF treatment. This approach creates medial and lateral windows within a single skin incision, allowing for simultaneous volar plating of the DFF. The lateral window is based on the modified Henry’s approach, while the medial window is created through the interval between the flexor carpi ulnaris (FCU) and the finger flexors. This technique aims to achieve rigid fixation in a minimally invasive, anatomical, and safer manner.

## 2. Materials and Methods

A retrospective study was performed of all patients who had undergone ORIF of a DFF at a single trauma center. Inclusion criteria comprised all skeletally mature patients who sustained a displaced DRF associated with a mal-aligned ulna head/neck fracture after fixation of the radius. Malalignment of a distal ulnar metaphyseal fracture is defined by an angular deformity of 10 degrees or more, a change in ulnar variance of 3 mm or greater, or a translation of the fracture surface by one-third or more [5]. The instability of a distal ulnar metaphyseal fracture is indicated by the movement of fracture fragments during passive forearm rotation. Similarly, DRUJ instability is characterized by the dislocation or subluxation of 50% or more of the distal ulna about the distal radius [16,19]. The exclusion criteria included nondisplaced ulna head fracture, isolated ulna styloid fracture, isolated ulna shaft fracture, severe comminution of the articular surface of the ulna head where screws cannot be securely placed, presence of arthritic changes in the DRUJ at the time of surgery, neurological involvement at the time of injury, and previous operations on the volar wrist, as these may affect anatomy or cause tissue adhesion. Between January 2020 and June 2023, 13 patients with DFFs underwent operative treatment involving osteosynthesis of both bones.

### 2.1. Classifications

In the study, patient fracture patterns were documented using two classification systems: the Biyani classification and the AO classification. Biyani et al. described a classification system for distal ulna metaphysis fractures, with or without associated ulnar styloid fractures. Type 1: simple extra-articular fracture with minimal comminution. Type 2: inverted T or Y-shaped fracture with an ulnar styloid fragment, including a portion of the metaphysis. Type 3: fracture of the lower end of the ulna with an avulsion fracture of the ulnar styloid. Type 4: comminuted fracture of the lower ulnar metaphysis, with or without a styloid fracture [12]. The AO classification of distal radius/ulna fractures is denoted as 2R3/2U3 and is further divided into detailed subtypes. Type A includes extra-articular fractures for ulna fractures: A1 for styloid process fractures, A2 for simple fractures, and A3 for multifragmentary fractures. Type B encompasses partial articular fractures, while Type C includes complete ones. For radius fractures, Type A includes extra-articular fractures: A1 for radial styloid avulsion fractures, A2 for simple fractures, and A3 for wedge/multifragmentary fractures. Type B encompasses partial articular fractures: B1 for sagittal fractures, B2 for dorsal rim fractures, and B3 for volar rim fractures. Type C includes complete articular fractures: C1 for simple articular and metaphyseal fractures, C2 for metaphyseal multifragmentary fractures, and C3 for articular multifragmentary fractures with simple or multifragmentary metaphyseal fractures [11].

### 2.2. Surgical Technique

We proposed a modified technique for DFF; via this approach, two windows are made for the volar plating of both bones with one wound incision: one is made based on the Modified Henry’s approach for DRF, and the other approach is made between the FCU and finger flexors for DUF.

The patient is positioned in supine position under general anesthesia. A tourniquet is set at 250 mmHg and placed on the upper limb, followed by having the skin disinfected and draped. The skin incisions are made between the palmaris longus (PL) and flexor carpi radialis (FCR). The incision is made longitudinally from the wrist distal flexion crease and extended proximally around 8 to 9 cm; the incision length depends on the fracture patterns. Incision of the tendon sheath of FCR retracts the tendon to the medial side. Then, deep dissection was carried out at intervals between the FCR and radial artery. Then, the pronator quadratus (PQ) is released on the most radial side of the radius with reverse L incision, and blunt dissection of the PQ is made until the ulnar side of the radius. Anatomic reduction of the radius fractures was achieved by minimal periosteum stripping and stabilized by the Acu-Loc 2/Acu-luc wrist plating system (Acumed, LLC., Hillsboro, OR, USA). This approach is based on the modified Henry’s approach to expose the radius, forming the lateral window of this new approach. The reduction quality of the radius was scrutinized under fluoroscopy, and the stability of the DRUJ and DUF was examined simultaneously.

**Figure 1:**
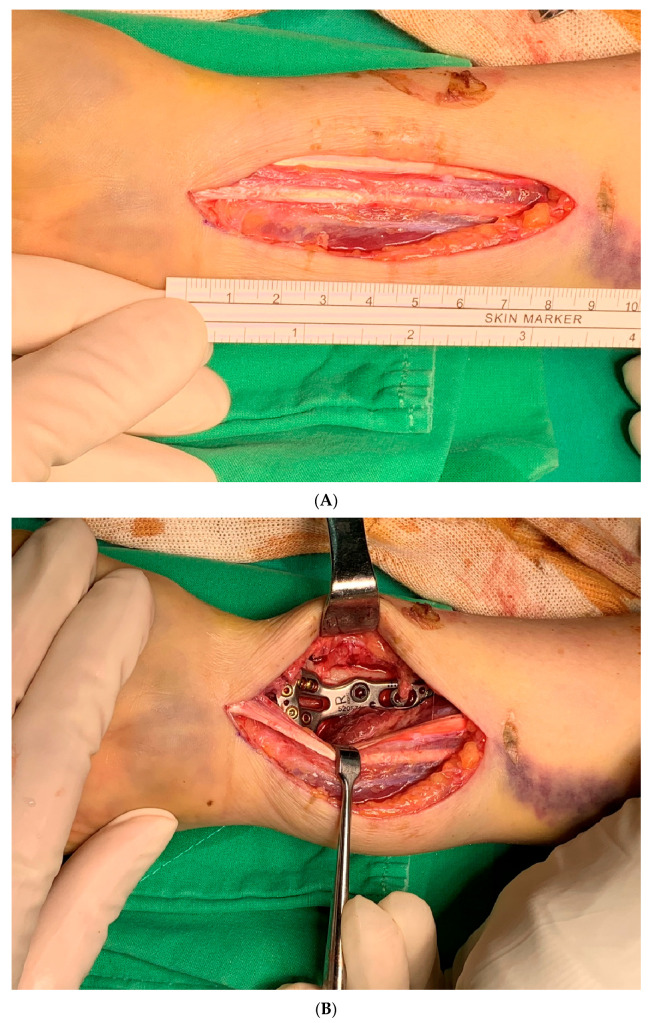

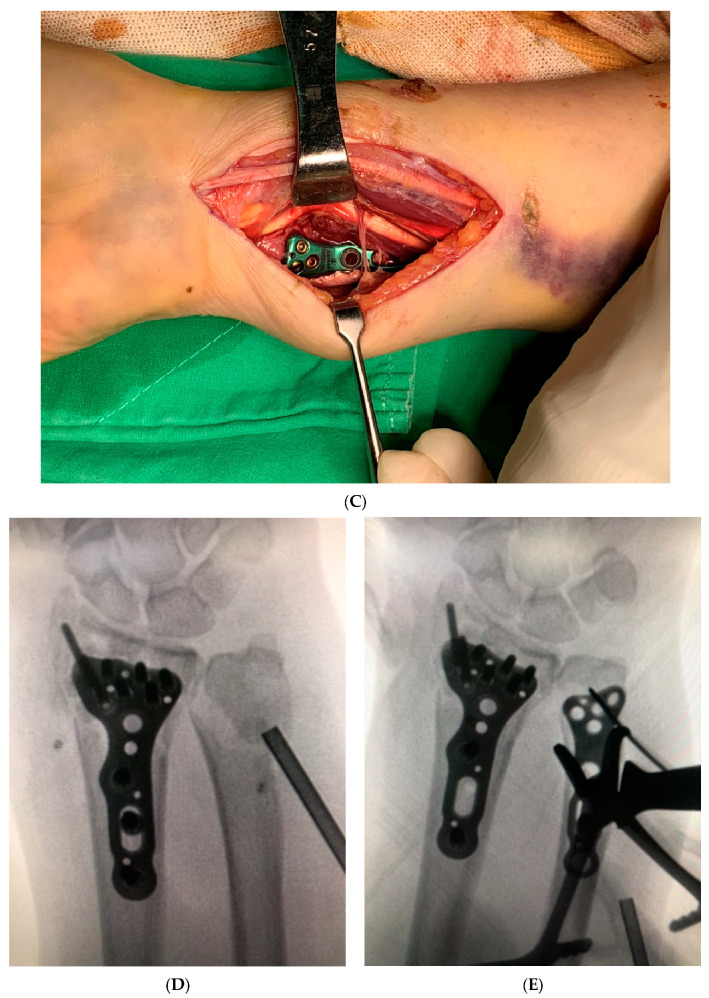
The figures showed intraoperative images of initial fracture fixation through the modified dual window approach. (**A**) Straight skin incision is made between the interval of flexor carpi radialis (FCR) and palmaris longus (PL), which is around 8–9 cm; (**B**) ORIF of the radius was conducted through the lateral window. (**C**) ORIF of the ulna was conducted through the medial window. (**D**) fluoroscopy: anteroposterior view after final fixation of the DRF; check the alignment of the DUF. (**E**) fluoroscopy: DUF under ORIF with a point-to-point reduction clamp; the skin incision is made between the interval of FCR and PL.

**Figure 2:**
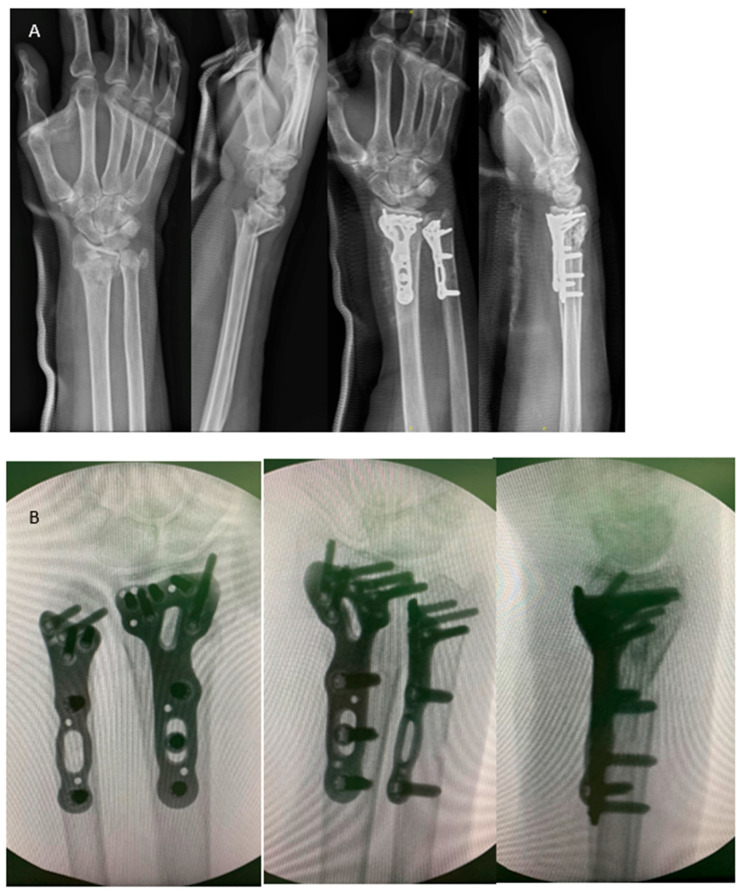
(**A**) A sixty-seven-year-old woman sustained a distal forearm fracture. She underwent ORIF to both bones via the modified dual channel approach. The figure shows anteroposterior/lateral view at initial and postoperative day 1. (**B**) Ensure the locking plate placement on the ulna does not extend beyond the radius sigmoid notch to avoid impingement. The three intraoperative fluoroscopic images, respectively, represent the AP view, PA view, and lateral view, which may help us identify the plate positioning and avoid screw intra-articular penetration.

**Figure 3:**
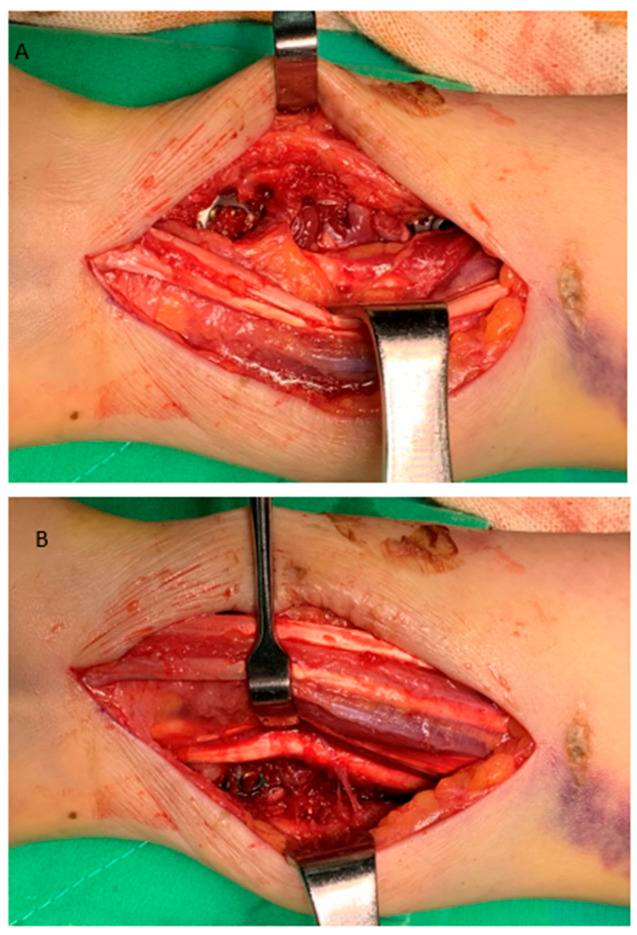
The figure shows the repair of the pronator quadratus muscle back to the ulna and then to the radius through two surgical windows. (**A**) lateral window, (**B**) medial window.

As for the medial window of our proposed approach, the dissection is processed crossly in the anterior subcutaneous layer to the other side of the median nerve by a blunt dissection. The surgeon should avoid damage to the palmar cutaneous branch of the median nerve (PCBMN), which was merged subcutaneously midway between the radial and ulnar styloid processes, with the mean position of this emergence 5.7 mm proximal to the styloid line [20]. The medial window was created between the FCU and flexor digitorum superficialis (FDS). The dissection proceeded between the flexor digitorum profundus (FDP) and the ulnar neurovascular bundle. After that, the PQ is partially released along its ulnar attachment, depending on the fracture pattern. Therefore, anatomic reduction of the ulna fracture was achieved by ORIF with the Acu-Loc 2/Acu-luc wrist plating system (Acumed, LLC., Hillsboro, Oregon, USA) (Figure 1, Figure 2 and Figure 3).

### 2.3. Postoperative Management

After wound closure in layers, a protected short arm splint is recommended for two weeks. Patients are encouraged to perform finger pumping exercises and range of motion activities for the elbow. Once the short arm splint is discarded, patients should begin wrist exercises such as flexion, extension, forearm pronation, supination, and grip strengthening exercises under a hand therapist for at least three months.

### 2.4. Clinical Evaluation

All clinical and functional outcomes were evaluated monthly for the first three months after surgery and subsequently every three months for the following one year. We collected the following data: the visual analog score during activity, the range of motion and arc angle of the injured wrist, the percent difference in the grip strength between the injured wrist and the uninjured wrist, and the Quick Disabilities of the Arm, Shoulder, and Hand (Q-DASH) score. Grip strength assessment was performed using a dynamometer (Smedley’s dynamometer, TTM, Tokyo, Japan) with patients sitting upright. The shoulder was adducted to the body, the elbow extended, and the wrist in a neutral position. Data were collected twice for each test, with the higher values included in the study. The best Q-DASH score is 0%, and the worst is 100%. Postoperative radiographic parameters, such as radial height, inclination, volar tilt, and ulnar variance were recorded. A final evaluation was conducted one year after surgery. All data in the study were presented using descriptive statistics, specifically as means and standard deviations. Postoperative complications, such as surgical site infections, discomfort, pain due to the implant impingement followed by implant removal, iatrogenic nerve injury, and complex regional pain syndrome, were recorded during the follow-up period.

## 3. Results

Thirteen patients with a DFF treated operatively were included. There were eight females and five males with a mean age of 52.3 ± 18.3 years (range 20 to 73 years). The mean follow-up was 12.1 ± 3.2 months (range 4 to 18 months). Demographic data and fracture classifications are listed in Table 1.

**Figure 4:**
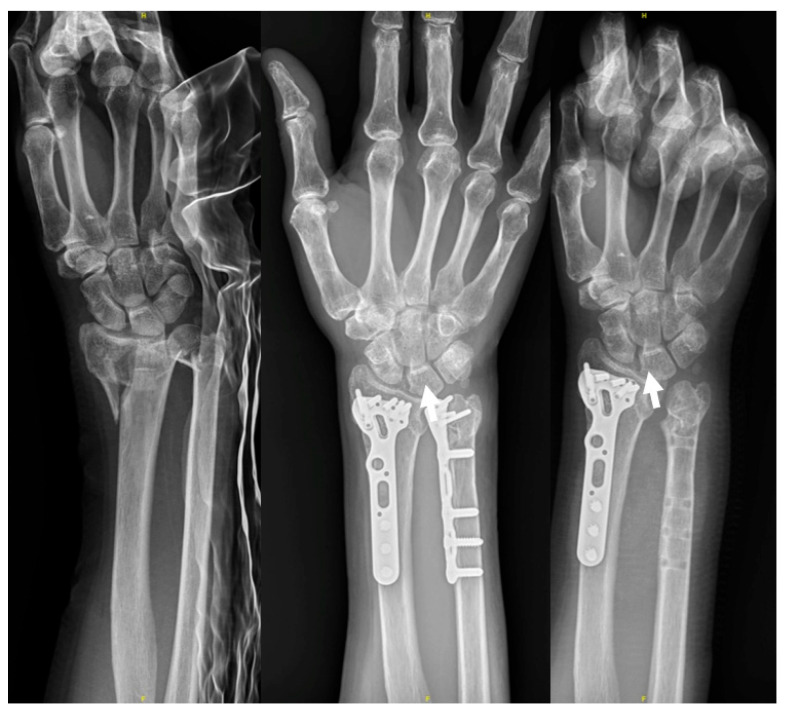
The figure shows a right distal forearm comminuted fracture pre- and post-operatively at nine months. The ulna implant was removed due to irritation, which caused bony erosion at the DRUJ. The white arrow indicates the erosion near the sigmoid notch.

At the last follow-up, wrist flexion was 67 ± 11° and extension was 69 ± 13°. Forearm pronation was 81 ± 9° and supination was 79 ± 15°. Grip strength was 28.3 ± 11.5 kg, which was 88% of the uninjured opposite side. The mean Quick DASH score was 13.9 ± 11.5. The mean VAS during activity was 0.5 ± 0.9. The postoperative radiographic parameters were as follows: radial height: 10.8 ± 1.7 mm, radial inclination: 22.6 ± 3.7°, volar tilting: 4.0 ± 3.9°, and ulnar variance: −0.4 ± 1.4 mm. There were no cases of infection, neurovascular injury, or malreduction among these patients. However, impingement on the sigmoid notch of the radius caused by the ulnar implant, necessitating implant removal, was reported in two patients (15.4%) (Figure 4). Clinical results and radiographic parameters are summarized in Table 2.

## 4. Discussion

Distal ulnar metaphyseal fractures occur from the ulnar neck to within 5 cm of the distal dome of the ulnar head [21]. These fractures are uncommon in isolation and are typically associated with DRF. Ulnar-sided wrist injuries often occur alongside DRF. It is crucial to consider the integrity and stability of the DRUJ, which may be compromised by tears of the triangular fibrocartilage complex (TFCC) or malunion of the DFF [22,23,24]. When a DUF heals in malunion, bidirectional instability of the DRUJ can arise. An associated injury to the TFCC often compounds this instability, as the deformity compromises the distal interosseous membrane’s role as a secondary stabilizer [25]. When managing DFFs, it is essential to address the DRF before considering the treatment of a distal ulna metaphyseal fracture. If a distal ulnar metaphyseal fracture is properly realigned and stabilized following the reduction and fixation of the radius fracture, cast treatment can be effectively utilized for the ulnar fracture [5,21]. Furthermore, there are currently insufficient evidence or data to establish a definitive treatment guideline for distal forearm fractures, specifically regarding whether osteosynthesis of the radius alone is sufficient or if simultaneous osteosynthesis of both bones is necessary. Most studies have focused on the elderly, and there is a lack of large-scale studies providing evidence for relatively younger patients.

The study presents a modified surgical approach that offers improved exposure of the DFF compared to conventional double incision methods, such as the modified Henry’s approach for DRF and the lateral approach for DUF. This approach is particularly advantageous for cases involving distal ulna comminution or DRUJ involvement. This approach enhances the surgical field’s visualization and improves fixation efficacy. It enables direct access to both bones simultaneously, allowing the quality of fixation to be assessed without frequent changes in forearm position, as required with the double incision method. The thin, soft tissue coverage and the ridge along the ulna make locking plate placement more challenging and uncomfortable for patients, potentially necessitating implant removal. A recent study showed that ulna hook plate osteosynthesis for DUFs via a direct lateral approach in young and active patients allows for solid fixation and a quick return to daily activities. However, complications such as DCBUN injury or implant irritation are common and often require implant removal [26].

Fractures of the distal ulnar neck or head often involve small fragments, making fixation challenging, particularly by direct lateral approach. This difficulty is exacerbated in elderly patients who have comminuted or osteopenic bones. Various DUF fixation methods have been described, including K-wires, tension band wiring, intramedullary devices, and plate-screw constructs [16,27]. Most surgeons choose implants based on their experience and implants availability. Locking-compression plates are typically used as internal fixators for DFFs due to their superior mechanical construct capable of withstanding deforming forces, even when only two to three screws are used to secure the distal fragments.

Several studies have analyzed the outcome of the operative treatment of DUF. Ring et al. reported that condylar blade plate fixation provides good alignment and satisfactory functional outcomes with an acceptable rate of secondary surgeries [5]. Han et al. indicated that seventeen patients were treated with LCP distal ulna plates for DUFs. All patients achieved bony union, with six cases rated as excellent and eleven as good according to Sarmiento’s modified wrist score at the 15-month follow-up. Therefore, fixation of the DUF is a recommended implant option for unstable DFFs [28]. Dennison’s research focused on the effectiveness of locking plate fixation for unstable DUFs occurring alongside DRFs. His study involved five patients, each treated with 2.0 mm mini-locking plates for their unstable DUF. The outcomes were notably positive: all fractures achieved union, with an average wrist range of motion of 59 degrees in flexion and extension, 67 degrees in pronation, and 72 degrees in supination. Despite the study’s small sample size and short follow-up, the findings were promising, with all cases showing excellent or good clinical outcomes [16]. A study reported a 6% incidence of associated metaphyseal distal ulna fractures in 320 distal radius fracture patients. Among the 19 patients who underwent nonsurgical management of the ulna, six had fair or poor results, and seven experienced limited forearm rotation [24]. Providing stable fixation for the DFF aims to minimize complications and preserve the DRUJ integrity and the wrist and forearm functionality [12,29,30]. The dual window approach raises concerns about the increased dissection of the pronator quadratus muscle and potential residual wrist weakness. This study demonstrated slightly superior clinical results compared to previous studies. The Q-DASH score was 13.9 ± 11.5. The average range of motion for wrist flexion, extension, forearm pronation, and supination was around 80% to 90% compared to the contralateral side, and grip strength was approximately 88% of the contralateral side.

Given the complexities of fixing distal metaphyseal ulnar fractures, numerous studies have demonstrated the effectiveness of conservative treatment options as valid alternatives. Cha et al. conducted a prospective study comparing two approaches for treating unstable DUFs that occur alongside DRFs in individuals over 65. In both groups, the DRFs were addressed using internal fixation. Their findings showed no notable differences between the two groups’ clinical or radiological results. Interestingly, none of the patients developed symptomatic arthritis in the radiocarpal or DRUJ by the final follow-up. This study indicates that conservative treatment is viable for managing DFFs in the elderly [14]. Liang et al. highlighted the critical role of soft tissue stabilizers, especially the TFCC and the distal oblique band (DOB) of the interosseous membrane, in ensuring distal forearm stability. Their research on DFFs revealed that accurate reduction of the distal radius is essential for reinstating the structural integrity of the DOB and TFCC. They concluded that this restoration is vital for preserving the reduction of distal ulnar metaphyseal and articular fractures [1]. Kurozumi et al. aimed to determine if a simultaneous fixation of the distal radius and distal ulnar fractures improves outcomes. Twenty-three patients treated for DRFs over four years were studied; 14 ulnar fractures were surgically fixed, and nine were treated conservatively. Outcomes measured included range of motion, grip strength, ulnar variance, bone union, and Q-DASH scores. There was no significant difference in Q-DASH scores between the groups, though the operative group had more restricted dorsi-palmar flexion. They concluded that conservative management is effective for DUFs when the DRF is fixed correctly, indicating no benefit from simultaneous fixation [31]. Most of the studies were not randomized, and the decision to fixate the ulna was based on the surgeon’s experience. Otherwise, there is a tendency to surgically fix more displaced or unstable DUFs, while less displaced or severe fractures are managed non-surgically. This could result in outcomes influenced by selection bias. More systematic and evidence-based guidelines on when and how to fix DUFs need to be developed.

A systemic review indicated that 17 studies compared the complications and reoperations following nonoperative treatment (209 fractures), ORIF (237 fractures), and distal ulnar resection (66 fractures). ORIF had the overall complication rate of 14.3 ± 21.3% and reoperation rate of 9.3 ± 10.5%. The most common complications included nonunion, ulnar-sided pain, and ulnar nerve palsy. The primary reasons for secondary operations were implant removal (6.3%) and revision ORIF (2.1%) [18]. Our study recorded no complications such as neurovascular issues, nonunion, malreduction, or infections. However, two patients underwent implant removal due to implant impingement during follow-up. This proposed technique involves placing the plate on the volar side for comfort considerations. Placing hardware along the volar surface of the ulna is more manageable and better tolerated due to its broader, flatter surface and thicker soft tissue envelope [32]. Dennison et al. mentioned that patients tolerated the plate well, possibly due to the relatively low profile of the implant when positioned more volar [16]. Surgeons must take care to avoid impingement on the sigmoid notch during forearm rotation if the plate is positioned too distally while dealing with comminuted fractures.

Mares et al. reported a similar approach using a fixed-angle locking plate for DRF in 2012. This method was employed in 15 cases with satisfactory outcomes without PCBMN injury. However, radiographic evidence of union or functional outcomes was not mentioned. They suggested that an ulnarly curved incision in the distal aspect may avoid injury to the PCBMN [33]. The PCBMN runs distally between the FCR and the PL tendon, piercing the antebrachial fascia from deep to superficial, 1.5 cm distal to the scaphoid tubercle [34]. Our study utilized a straight incision, slightly ulnar to the FCR tendon, without reported median nerve injuries. The median nerve and its palmar cutaneous branch were effectively protected by meticulously dissecting without crossing the bistyloid line—where the PCBMN emerges distally—and navigating between the subcutaneous tissue and superficial fascia. This technique helps avoid damage to the PCBMN and avoid traction injury to the median nerve and its branches. Additionally, it provides good access to the lunate facet with attached radiocarpal ligament are crucial for stabilizing the wrist joint. The proposed dual window with single incision approach allows the surgeon to manipulate the median nerve bundle in either the ulnar or radial direction, obtain a more extensive surgical field, and achieve more easily anatomic reduction.

This study has several limitations. Firstly, the small sample size of thirteen patients limits the generalizability of the findings regarding DFFs. Secondly, the current fracture classification system does not consider factors like displacement degree or fracture severity, such as periosteal stripping or articular surface impaction. As a result, these variables were not discussed separately. Thirdly, as a retrospective study, it reflects the outcomes of two experienced surgeons. Due to increased complications like malreduction, implant-related issues, and nerve injury with the conventional method for DFF, fewer surgeries are performed using this method, leading to the absence of a control group. Additionally, there is no information on the patients’ bone quality. Future studies should include DEXA scans to determine the suitability of ORIF for distal ulna fractures and to explore any correlation between postoperative outcomes and the patients’ DXA results. A larger-scale, well-designed prospective study will be conducted in the future to address these limitations and enhance the validity of the findings.

## 5. Conclusions

A volar dual window with a single incision approach is proposed for treating DFFs. The study demonstrates that this approach can be successfully implemented with minimal invasiveness. Osteosynthesis of unstable DFFs using this method may result in excellent alignment, good function, and comparable grip strength. This approach may help reduce casting immobilization time, and decrease the likelihood of subsequent revision surgery. The results indicate that this modified approach is relatively safe and offers equivalent functional recovery and reduction quality for DFF, with fewer complications than the traditional double incision approach.

## Figures and Tables

**Table 1:** Demographic data and fracture classifications.

	Patients (*n* = 13)
Sex	8 females, 5 males
Age (years)	52.3 ± 18.3
Follow-up (months)	12.1 ± 3.2
AO/OTA classification	numbers
Radius (2R3)	
A2	1
1	1/1
1/1	2/6/2
2/6/2	
A2/A3	9/3
C	1
Biyani classification (ulna)	
1	4
2	2
3	6
4	1

**Table 2:** Clinical results, radiographic parameters, and postoperative complications at final follow-up.

	Patients (*n* = 13)
Clinical scores	
Q-DASH score	13.9 ± 11.5
Visual analog scale during activity	0.5 ± 0.9
Grip strength (kg)	
Injured hand	28.3 ± 11.5
Healthy hand	32.2 ± 8.6
Range of motion (°)	
Wrist flexion	67 ± 11
Wrist extension	69 ± 13
Forearm pronation	81 ± 9
Forearm supination	79 ± 15
Radiographic parameters at final follow-up	
Radial height (mm)	10.8 ± 1.7
Radial inclination (°)	22.6 ± 3.7
Volar tilting (°)	4.0 ± 3.9
Ulnar variance (mm)	−0.4 ± 1.4
Postoperative complications	
Implant removed due to impingement of DRUJ	2 patients (15.4%)

## Data Availability

The data presented in this study are available on request from the corresponding author. The data are not publicly available due to local institutional ethics board regulations.

## Abbreviations

ORIF: open reduction and internal fixation; DFF: distal forearm fracture; DRF: distal radius fracture; DUF: distal ulna fracture; DRUJ: distal radioulnar joint; DCBUN: dorsal cutaneous branch of the ulnar nerve; PCBMN: palmar cutaneous branch of the median nerve; TFCC: triangular fibrocartilage complex; Q-DASH: Quick disabilities of the arm, shoulder and hand; VAS: Visual analog scale.
